# Population-level adult mortality following the expansion of antiretroviral therapy in Rakai, Uganda

**DOI:** 10.1080/00324728.2019.1595099

**Published:** 2019-05-23

**Authors:** Dorean Nabukalu, Georges Reniers, Kathryn A. Risher, Sylvia Blom, Emma Slaymaker, Chodziwadziwa Kabudula, Basia Zaba, Fred Nalugoda, Godfrey Kigozi, Fred Makumbi, David Serwadda, Steven J. Reynolds, Milly Marston, Jeffrey W. Eaton, Ron Gray, Maria Wawer, Nelson Sewankambo, Tom Lutalo

**Affiliations:** 1 Rakai Health Sciences Program; 2 London School of Hygiene and Tropical Medicine; 3 University of the Witwatersrand; 4 Makerere University; 5 National Institutes of Health; 6 Johns Hopkins Bloomberg School of Public Health; 7 Imperial College London; 8 Uganda Virus Research Institute

**Keywords:** antiretroviral therapy (ART), HIV, mortality, Rakai, Uganda

## Abstract

There are limited data on the impact of antiretroviral therapy (ART) on population-level adult mortality in sub-Saharan Africa. We analysed data for 2000–14 from the Rakai Community Cohort Study (RCCS) in Uganda, where free ART was scaled up after 2004. Using non-parametric and parametric (Weibull) survival analysis, we estimated trends in average person-years lived between exact ages 15 and 50, per capita life-years lost to HIV, and the mortality hazards of people living with HIV (PLHIV). Between 2000 and 2014, average adult life-years lived before age 50 increased significantly, from 26.4 to 33.5 years for all women and from 28.6 to 33.8 years for all men. As of 2014, life-years lost to HIV had declined significantly, to 1.3 years among women and 0.4 years among men. Following the roll-out of ART, mortality reductions among PLHIV were initially larger in women than men, but this is no longer the case.

## Introduction

Before the introduction of antiretroviral therapy (ART), HIV/AIDS was a leading cause of death among adults aged 15–49 years in the rural Rakai district, Uganda. Among adults with HIV, mortality in the 1990s was around 132.6 deaths per 1,000 (Serwadda et al. 1985; Sewankambo et al. 1994, 2000). Many clinical cohort studies have demonstrated the impact of ART on adult mortality in ‘people living with HIV’ (referred to hereafter as PLHIV) who are enrolled in treatment programmes (Herbst and Cooke 2008; Brinkhof et al. 2009; Mills, Bakanda, Birungi, Chan, Ford, et al. 2011), but have not assessed the impact of ART at a population level. Demographic surveillance data are needed to estimate population-level effects of ART because such data include PLHIV who are not in care. A number of studies have documented the impact of ART on population-level mortality trends in eastern and southern Africa (Jahn et al. 2008; Herbst et al. 2009; Kasamba et al. 2012).

Some of these studies have estimated the life-years gained following the scale-up of ART and the number of life-years that remain lost to HIV (Bor et al. 2013; Asiki et al. 2016; Price et al. 2017; Reniers et al. 2017). These studies report better outcomes for women than men due to higher ART use (Muula et al. 2007; Druyts et al. 2013; Staveteig et al. 2013), earlier treatment initiation, and lower attrition and mortality while on ART among women (May et al. 2010; Hawkins et al. 2011; Mills, Bakanda, Birungi, Chan, Hogg, et al. 2011; Cornell et al. 2012; Auld et al. 2013; Druyts et al. 2013).

In this study we use data from the Rakai Community Cohort Study (RCCS), a demographic and HIV surveillance cohort study in the rural Rakai District of south-central Uganda, to estimate the population-wide impact of ART on adult mortality.

## Methods

Since 1994 the Rakai Health Sciences Program (RHSP) has conducted an open, community-based cohort study, the RCCS. All households in 50 communities were enumerated and, within one month, consenting individuals aged 15–49 years were enrolled in an HIV survey. The design and conduct of the RCCS have been described elsewhere (Wawer et al. 1998, 1999). In this analysis, we include 30 communities that were consistently followed from 2000 to 2014.

Information from the household census was used to update the residency and vital status of household members, and the individual interviews were used to elicit greater detail on the risk factors for a variety of health outcomes, including HIV. Survey participants provided a venous blood sample for HIV detection using two enzyme immunoassays, with western blot or polymerase chain reaction confirmation, or both. To allocate exposure time to HIV status categories, we classified person-time before the first recorded HIV test as ‘HIV status unknown’. Person-time following a positive HIV test remained ‘HIV-positive’ for the duration until censoring by loss to follow-up, end of study, or death. Exposure time between two negative tests was counted as ‘HIV-negative’, and the time following an individual's last negative test was also considered HIV-negative for an estimated duration of time based on the probability that 95 per cent of their age group remained uninfected, given sex-specific HIV incidence rates.

### Ethical considerations

Before enrolment into the RCCS, all adult participants (aged 18–49) provided written informed consent. Adolescents (aged 15–17) provided assent and their parents or guardians provided consent on their behalf. The RCCS was approved by the Uganda Virus Research Institute's Science and Ethics Committee (currently the Research Ethics Committee), the Uganda National Council for Science and Technology, Institutional Review Boards (IRBs) at Johns Hopkins and Columbia universities, and Western IRB in the United States (US).

### HIV care programme

Until 2004, there was limited access to ART in Uganda (Wendo 2004). In June 2004, with funding from the US President's Emergency Plan for AIDS Relief (PEPFAR), RHSP started to provide free HIV treatment in Rakai district in accordance with World Health Organization (WHO) ‘CD4’ criteria for treatment initiation. Through record linkage with RHSP treatment facilities, we ascertained the treatment status of RCCS participants living with HIV using unique identifiers. However, there are other medical services in the study area whose data could not be linked to the RCCS. The RHSP had provided HIV care to a total of 20,909 HIV clients in the district as of December 2014. Of these, 7,436 were RCCS participants (around 52 per cent of the HIV-positive RCCS participants), of which 6,618 (around 89 per cent) were known to be on ART as of December 2014. Enrolment into HIV care and the ART programme is based on the WHO ART eligibility criteria and the Ugandan National Antiretroviral Treatment Guidelines (Katabira et al. 2009). In this analysis, we distinguish between the ‘pre-ART period’ (January 2000–May 2004) and the ‘ART period’ (June 2004–December 2014).

### Statistical analysis

We estimated person-years lived as the area under the Kaplan–Meier survival curve between exact ages 15 and 50, by calendar year, HIV status, and sex (Kalbfleisch and Prentice 2002). This measure, sometimes referred to as partial life expectancy (Hickman and Estell 1969), can be interpreted as the population-average number of years that a 15-year-old lives up to their 50th birthday under the prevailing mortality conditions. The maximum value of 35 years would be reached if there were no mortality in the age range under consideration (Kaplan and Meier 1958). We used the measure of years lived between 15 and 50 instead of adult life expectancy because individuals in the RCCS are not followed beyond age 50. Life-years lived in adulthood is a useful measure to track the impact of ART on HIV-associated mortality, as it is independent of the population age structure. We also estimated the number of life-years lost to HIV. This was computed as the difference between the life-years lived by known HIV-negative individuals and the life-years lived by the whole population. In other words, the years lived by HIV-negative individuals serves as a benchmark, and the shortfall in the population-average number of years lived quantifies the residual burden of HIV mortality in this population (Kalbfleisch and Prentice 2002; Klein and Moeschberger 2003; Kleinbaum and Klein 2012). Percentile-based confidence bounds for the number of life-years lived and lost were obtained via bootstrapping with 1,000 replications. A comparable approach has been used in other studies (Bor et al. 2013; Asiki et al. 2016; Price et al. 2017; Reniers et al. 2017).

We next examined mortality trends among PLHIV by treatment status, using parametric survival analysis to model the mortality hazards among PLHIV, with age as the measure of time. The Weibull model was chosen over other models on the basis of model fit, and it yielded similar estimates of hazard ratios to those obtained with a semi-parametric model. We disaggregated survival analyses into the pre-ART and ART periods, and by treatment status, to differentiate PLHIV who had never initiated ART from those who had ever started ART (even if it was interrupted or stopped). We considered sex, calendar year, and their interaction as covariates. Data before the introduction of ART in the study communities (January 2000–May 2004) provided an estimate of baseline (pre-ART) mortality. Given the age-dependent covariate calendar year (*y*(*t*)) and the age-independent covariate sex (*s*), the mortality hazard in the pre-ART period is estimated by:

(1) $$h(t|y(t),s)=pt^{p-1}exp\{b_{0}+b_{1}y(t)+b_{2}s+b_{3}s\times y(t)\}$$

where *t* is age and *p* is the Weibull shape parameter which yields a monotonically increasing mortality hazard for values greater than one (Kalbfleisch and Prentice 2002; Klein and Moeschberger 2003; Kleinbaum and Klein 2012). In the ART period, the mortality hazard is estimated as:

(2) $$\begin{array}{rl} h(t|y(t),s) & =pt^{p-1}exp\{b_{0}+b_{1}(y(t)-2003{)}^{+}\\ & \;+b_{2}(y(t)-2008{)}^{+}+b_{3}s+b_{4}s\\ & \;\times (y(t)-2003{)}^{+}+b_{4}s\times (y(t)-2008{)}^{+}\} \end{array}$$

where the spline term (*y*(*t*) − 2003)*^+^* is equal to zero if *y*(*t*) ≤ 2003, equal to *y*(*t*) − 2003 if 2004 ≤ *y*(*t*) < 2008, and equal to 2008 − 2003 if *y*(*t*) ≥ 2008. Similarly, the spline term (*y*(*t*) − 2008)*^+^* is equal to zero if *y*(*t*) ≤ 2008, and equal to *y*(*t*) − 2008 if *y*(*t*) > 2008. Across all models, to demonstrate changes in calendar time among men, linear combinations of the calendar time (*y*(*t*)) and sex × calendar time interaction (*s* × *y*(*t*)) coefficients are presented.

## Results

Between 2000 and 2014 the RCCS enumerated 58,871 individuals aged 15–49, who contributed a total of 183,581 person-years of observation (pyo) and 1,374 deaths (Table 1). A higher number of women (31,476) than men (27,395) participated in the RCCS, including 3,039 women and 1,514 men who tested HIV-positive during the study period. The low age cut-off in part explains the relatively small number of HIV-positive men in the data set, because the age profile of HIV-positive men is usually older than that of HIV-positive women (Gregson et al. 2002). Among PLHIV, at least 708 women (23.3 per cent) and 311 men (20.5 per cent) initiated ART between June 2004 and December 2014 (inclusive).

**Table 1:** Person-years, deaths, and mortality rates by sex and treatment period, ages 15–49, Uganda 2000–14

Variables	Pre-ART period (2000–03)^1^		ART period (2004–14)^2^	
	Male	Female	Male	Female
Number of individuals	9,396	10,776	17,999	20,700
Number of person-years	20,924	22,822	67,403	72,432
Number of deaths	249	313	432	380
Number of deaths to HIV-positive individuals	125	224	185	193
*Mortality rates (per 1,000 pyo)*				
Overall death rate (all-cause)	11.9 (10.5–13.5)	13.7 (12.3–15.3)	6.4 (5.8–7.0)	5.2 (4.7–5.8)
Death rate for HIV-negative individuals	3.1 (2.2–4.3)	2.3 (1.6–3.3)	3.0 (2.4–3.6)	2.2 (1.8–2.7)
Death rate for HIV-positive individuals (PLHIV)	101.0 (84.7–120.3)	100.4 (88.1–114.4)	43.9 (38.0–50.7)	24.1 (20.9–27.7)
Death rate for individuals with HIV status unknown	10.6 (8.6–13.0)	8.5 (6.6–11.1)	5.1 (4.3–6.0)	4.3 (3.5–5.2)

^1^The period 2000–03 includes January–May 2004.

^2^The period 2004–14 excludes January–May 2004.

*Note*: 95 per cent confidence intervals are shown in parentheses. Pyo refers to person-years of observation.

*Source*: Authors’ calculations from RCCS data.

**Figure 1:**
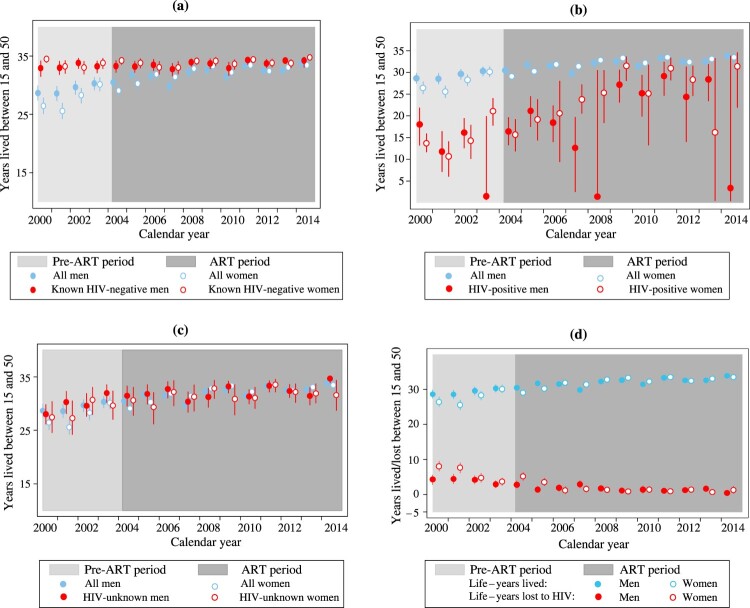
Life-years lived by (a) HIV-negative individuals, (b) HIV-positive individuals, and (c) individuals with unknown HIV status, compared with the population as a whole; and (d) population-wide life-years lived and lost to HIV; all between ages 15 and 50, by sex and calendar year, Uganda 2000–14 *Source*: Authors’ calculations from RCCS data.

Following the roll-out of ART in 2004, the overall death rate in adulthood decreased from 11.9 deaths (95 per cent confidence interval (CI): 10.5–13.5) to 6.4 deaths per 1,000 pyo (95 per cent CI: 5.8–7.0) among men, and from 13.7 deaths (95 per cent CI: 12.3–15.3) to 5.2 deaths per 1,000 pyo (95 per cent CI: 4.7–5.8) among women. The mortality decline among PLHIV was more pronounced among women: adult mortality rates in HIV-positive men declined from 101.0 (95 per cent CI: 84.7–120.3) to 43.9 deaths per 1,000 pyo (95 per cent CI: 38.0–50.7), whereas those of HIV-positive women declined from 100.4 (95 per cent CI: 88.1–114.4) to 24.1 deaths per 1,000 pyo (95 per cent CI: 20.9–27.7) (Table 1). These mortality declines translated to substantial increases in the life-years lived in adulthood (partial life expectancy between ages 15 and 50; see Figure 1). The population-wide number of life-years lived between ages 15 and 50 (Figure 1(a), all men and women) increased by 7.1 years for women, from 26.4 (95 per cent CI: 25.1–27.9) to 33.5 years (95 per cent CI: 32.2–34.5), and by 5.2 years for men, from 28.6 (95 per cent CI: 27.4–29.9) to 33.8 years (95 per cent CI: 32.9–34.7). It is worth noting that partial life expectancy had started to increase before the roll-out of ART, in part due to the use of prophylaxis with cotrimoxazole.

Among HIV-negative individuals there was minimal mortality in early adulthood, and the average number of years lived between ages 15 and 50 approached the ceiling of 35 years for both sexes, with minimal change over time (years lived ranged from 32.7 to 34.7 years; Figure 1(a)). Among PLHIV (Figure 1(b)), the years lived in adulthood increased by more than ten years for both men and women following the introduction of ART (see the 2004–14 period for women and 2004–13 for men), but confidence intervals are wide because of small numbers. In addition, the estimates for PLHIV are sensitive to the timing of diagnosis or HIV testing, which complicates their interpretation.

The increase in life-years lived for those of unknown HIV status was similar to that seen in the total population, which suggests that individuals whose HIV status was unknown are not highly selected with respect to HIV status (Figure 1(c)).

The life-years deficit, that is, the difference in the partial life expectancy between HIV-negative individuals and the population as a whole, is a more stable measure of the burden of HIV mortality at the population level (Figure 1(d)). For the year 2000, this burden was estimated at 8.1 years for women (95 per cent CI: 6.5–9.5) and 4.3 years for men (95 per cent CI: 2.9–5.7). By 2014, the adult life-year deficits in adulthood had almost entirely dissipated, to 1.3 years (95 per cent CI: 0.3–2.5) for women and 0.4 years (95 per cent CI: −0.4–1.3) for men.

**Figure 2:**
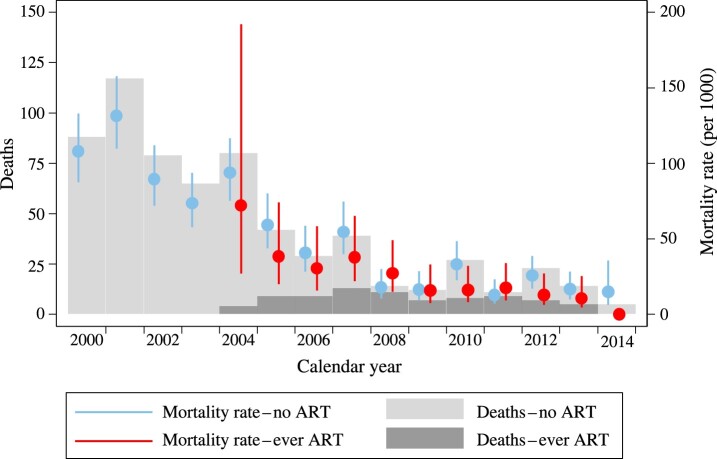
Trends in the total number of deaths among PLHIV (left-hand axis) and all-cause mortality rates for PLHIV (right-hand axis), by treatment status, Uganda 2000–14 *Source*: As for Figure 1.

Figure 2 illustrates trends in the number of deaths among PLHIV and their crude (all-cause) mortality rates by treatment status. Before the roll-out of ART in 2004, the mortality rates of PLHIV ranged from 74 to 132 deaths per 1,000 pyo. The first clear indications of a mortality decline among PLHIV are visible in 2005, and by 2008 mortality rates had dropped below 50 deaths per 1,000 pyo, both among those who had initiated treatment and among those for whom we do not have a record of treatment initiation. Mortality continued to decline, albeit more slowly, in the later years, decreasing in 2013 to 16.8 deaths per 1,000 pyo (95 per cent CI: 9.9–28.3) among PLHIV without a record of treatment initiation and to 10.5 deaths per 1,000 pyo (95 per cent CI: 4.4–25.3) among PLHIV who had ever started ART.

The trend in the total number of deaths among PLHIV corresponds to their mortality rates. In the years preceding the introduction of ART, there were between 65 and 117 deaths per year among PLHIV. The number of deaths per annum started declining in 2005, and by 2014 the annual death count among PLHIV was around five.

**Figure A1:**
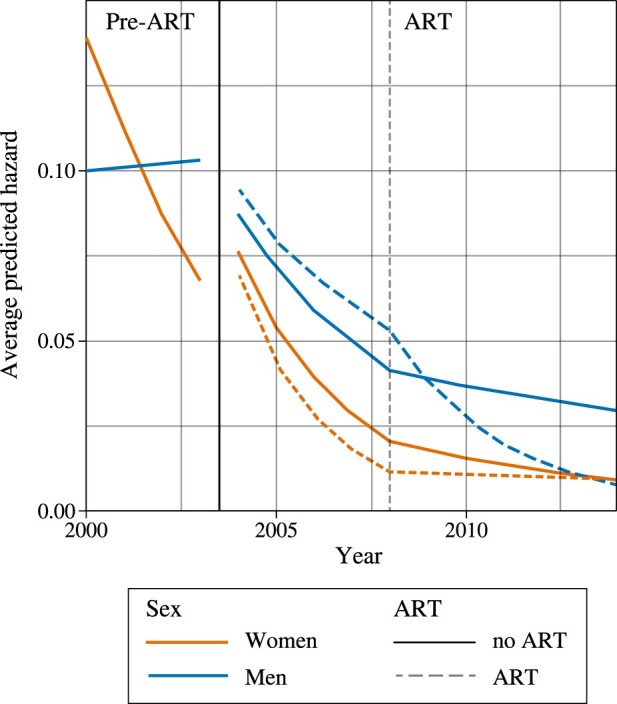
Model-predicted hazard of mortality for HIV-positive individuals by sex, calendar year, and ART status, Uganda 2000–14 *Note*: Dashed vertical line at 2008 represents the spline inflection in the model. *Source*: As for Figure 1.

Table 2 explores trends and sex differences in all-cause mortality among PLHIV, and average model-predicted mortality hazards are plotted in Figure A1. The first column of data in Table 2 shows results for the pre-ART period, while the second and third columns show the coefficients for the ART period. To allow for all two-way interactions with treatment status, the model was fitted separately for PLHIV who initiated treatment (the ‘treated’) and those for whom we do not have a record of treatment initiation (the ‘untreated’). In the pre-ART period, the coefficient for males is less than one and marginally significant, which suggests that the mortality rates for men living with HIV in the year 2000 may have been lower than those for women. However, this apparent mortality advantage among men quickly dissipated, as women exhibited a 22 per cent decline in mortality hazard per calendar year (hazard ratio (HR) = 0.78, 95 per cent CI: 0.69–0.88) alongside no discernible change in mortality risk across time for men (HR = 0.78 × 1.29 = 1.00, 95 per cent CI: 0.86–1.18) (Table 2). By 2003, the mortality risks for women had dropped below those of men (Figure A1). The large reduction in mortality among HIV-positive women in the pre-ART period may be partially attributable to small-scale roll-outs of ART in 2001–03 by the private sector or non-governmental organizations (NGOs) before RHSP treatments started in 2004 (Wendo 2004), which women may have benefited from more than men.

**Table 2:** All-cause mortality in HIV-positive individuals: hazard ratios and 95 per cent confidence intervals (Weibull regression), Uganda 2000–14

Variables^1^	Pre-ART (2000–03)^2^	ART (2004–14)^3^	
		Untreated	Treated
Calendar year (2000–03)	0.78	–	–
	(0.69–0.88)	–	–
Calendar year (2004–08)	–	0.72	0.64
	–	(0.67–0.77)	(0.57–0.71)
Calendar year (2009–14)	–	0.87	0.96
	–	(0.77–0.99)	(0.79–1.16)
Male^4^	0.65	0.93	0.93
	(0.45–0.94)	(0.76–1.14)	(0.75–1.16)
Male × calendar year (2000–03)	1.29	–	–
	(1.05–1.57)	–	–
Male × calendar year (2004–08)	–	1.15	1.36
	–	(1.04–1.28)	(1.17–1.58)
Male × calendar year (2009–14)	–	1.08	0.76
	–	(0.92–1.28)	(0.57–1.01)
Weibull shape parameter (*p*)	1.90	1.79	1.75
	(1.47–2.45)	(1.47–2.18)	(1.36–2.26)
Subjects	1,914	4,465	2,534
Deaths	349	645	431

^1^Estimates presented are hazard ratios from a Weibull regression model (with 95 per cent confidence intervals in parentheses). ‘Calendar year’ refers to a per-year relative hazard in the period specified.

^2^In the pre-ART analysis, the year 2000 is the baseline calendar year.

^3^In the ART analysis, we use 2000–03 as the baseline calendar year (including all individuals/episodes regardless of ultimate treatment status) for both untreated and treated analyses, and distinguish between untreated or treated episodes only from 2004–14. We do this to model the trend in mortality rates by treatment status from when ART was first introduced in Rakai.

^4^Reference group is females.

*Source*: As for Table 1.

In comparison with the pre-ART period, mortality decreased rapidly among PLHIV in the first decade of ART availability. For women the greatest decrease was observed in the first five years of ART availability (2004–08), while for men, the period in which the rate of decrease was larger depended on treatment status. During 2004–08, among untreated PLHIV, the risk of mortality decreased by 28 per cent per year for women (HR = 0.72, 95 per cent CI: 0.67–0.77) and by 17 per cent per year for men (HR = 0.72 × 1.15 = 0.83, 95 per cent CI: 0.77–0.89). The mortality risk among PLHIV who ever started ART decreased by 36 per cent per year among women (HR = 0.64, 95 per cent CI: 0.57–0.71) and by 13 per cent per year among men (HR = 0.64 × 1.36 = 0.87, 95 per cent CI: 0.78–0.96) in the first five years of ART availability.

From 2009 onwards, the risk of mortality decreased significantly for untreated women, by 13 per cent per year (HR = 0.87, 95 per cent CI: 0.77–0.99), but did not decrease for untreated men (HR = 0.87 × 1.08 = 0.94, 95 per cent CI: 0.84–1.05). After 2008, no significant decrease was observed in the mortality of women who ever initiated treatment (HR = 0.96, 95 per cent CI: 0.79–1.16), while that of men on ART decreased by 27 per cent per year (HR = 0.96 × 0.76 = 0.73, 95 per cent CI: 0.59–0.90).

The more rapid decrease in mortality in the first five years of ART availability among women reflects the fact that the earlier ART programme focused on treating the sickest individuals—those with lower CD4 counts or clinical AIDS—whereas the later programme initiated ART in asymptomatic individuals at higher CD4 counts.

## Discussion

This rural Ugandan population experienced important mortality reductions as a result of the expansion of ART programmes. In the year 2000, the per capita number of years lost to HIV in adulthood amounted to 8.1 years for women and 4.3 years for men. By 2014, ten years after the roll-out of free ART, the adult life-years deficits had shrunk to 1.3 and 0.4 years for women and men, respectively. Not all of the progress is directly attributable to ART, however, as evidenced by the fact that life-years lived in adulthood started increasing before ART became available in the study population. This finding corroborates that from another study in rural Uganda (Asiki et al. 2016). Because HIV incidence in Uganda peaked in the early 1990s, we would expect mortality to decline about ten years later (i.e., just before the roll-out of ART). In the RCCS, HIV incidence peaked in 2000 (at 1.3 per 100 pyo) and declined thereafter (Grabowski et al. 2017). This pattern contrasts with that seen in South Africa, which experienced a later epidemic and where the reversal in adult mortality did not occur until ART was introduced (Reniers et al. 2017).

Among PLHIV, mortality rates gradually declined after the roll-out of ART, both among those who had started ART, and among men and women for whom we did not have a record of treatment initiation. This is likely due to two complementary factors. First, some PLHIV may have received ART at health facilities whose records could not be linked to the demographic surveillance database. Second, the pool of PLHIV who had not yet started ART will have become increasingly healthier as a result of: (a) the changes in October 2011 to the treatment initiation thresholds from a CD4 cell count below 250 cells/μL to a count below 350 cells/μL; and (b) the adoption in September 2012 of the ‘Option B+’ programme for prevention of mother-to-child transmission, in which all HIV-positive pregnant women received ART for life, irrespective of their CD4 cell count.

Among PLHIV who had started ART, mortality initially declined faster among women, which corroborates earlier evidence that women's engagement with health services was better than that of men. Women are known to have higher HIV testing and counselling and higher ART coverage rates (Muula et al. 2007; Druyts et al. 2013; Staveteig et al. 2013), earlier treatment initiation, and lower attrition and mortality rates on ART (May et al. 2010; Hawkins et al. 2011; Mills, Bakanda, Birungi, Chan, Hogg, et al. 2011; Cornell et al. 2012; Auld et al. 2013; Druyts et al. 2013; Billioux et al. 2017). In more recent years, however, mortality among men with a record of ART initiation appears to have decreased more rapidly, which suggests that they are catching up.

Among PLHIV without a record of ART initiation, the initial sex differences in mortality were negligible, but the mortality risks declined faster for women than for men once ART became available. Again this serves as evidence that women engage with HIV services earlier than men, a phenomenon that may be reinforced by sex differences in HIV care and treatment eligibility as a result of the programmes for the prevention of mother-to-child transmission (Staveteig et al. 2013).

One important limitation of this study is that these analyses have an upper age cut-off of 50 years. Some HIV-associated mortality is likely to have occurred at ages above 50, and this is particularly the case for men, who are generally infected at older ages (Gregson et al. 2002). In other words, the analyses may underestimate the burden of HIV mortality among older men, as well as the mortality reductions this age group may have experienced.

## Conclusions

Important mortality reductions have been experienced by this rural Ugandan population. Mortality declines began before the introduction of treatment, as a result of historical declines in HIV incidence, and steadily declined thereafter as a result of the expansion of treatment programmes and treatment eligibility criteria. Mortality among PLHIV initially declined faster for women than for men, but the latter appear to be catching up. As of 2014, the residual burden of HIV mortality in adults under age 50 is estimated to be 1.3 life-years (95 per cent CI: 0.3–2.5) for women and 0.4 life-years (95 per cent CI: −0.4–1.3) for men.
